# Computational Study of Evolutionary Selection Pressure on Rainbow Trout Estrogen Receptors

**DOI:** 10.1371/journal.pone.0009392

**Published:** 2010-03-09

**Authors:** Conrad Shyu, Celeste J. Brown, F. Marty Ytreberg

**Affiliations:** 1 Department of Physics, University of Idaho, Moscow, Idaho, United States of America; 2 Department of Biological Sciences, University of Idaho, Moscow, Idaho, United States of America; Innsbruck Medical University, Austria

## Abstract

Molecular dynamics simulations were used to determine the binding affinities between the hormone 17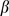-estradiol (E2) and different estrogen receptor (ER) isoforms in the rainbow trout, *Oncorhynchus mykiss*. Previous phylogenetic analysis indicates that a whole genome duplication prior to the divergence of ray-finned fish led to two distinct ER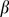 isoforms, ER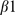 and ER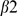, and the recent whole genome duplication in the ancestral salmonid created two ER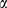 isoforms, ER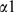 and ER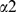. The objective of our computational studies is to provide insight into the underlying evolutionary pressures on these isoforms. For the ER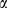 subtype our results show that E2 binds preferentially to ER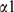 over ER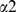. Tests of lineage specific 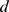
N/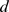
S ratios indicate that the ligand binding domain of the ER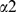 gene is evolving under relaxed selection relative to all other ER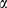 genes. Comparison with the highly conserved DNA binding domain suggests that ER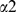 may be undergoing neofunctionalization possibly by binding to another ligand. By contrast, both ER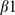 and ER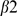 bind similarly to E2 and the best fitting model of selection indicates that the ligand binding domain of all ER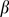 genes are evolving under the same level of purifying selection, comparable to ER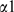.

## Introduction

Estrogens are essential endogenous hormones that modulate the development and homeostasis of a wide range of target tissues, such as the reproductive tracts, breast and skeletal system [1]. Estrogenic hormones have multi-faceted and wide-ranging effects in vertebrate animals. For estrogens such as 17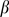-estradiol (E2) to exert their biological effects, they must interact with cellular estrogen receptors (ER). Studies have shown that ERs are part of two distinct estrogenic transduction pathways. One pathway provides a rapid, nongenomic pathway initiated by membrane bound ERs at the cell surface [1]–[3]. The other pathway provides direct genomic control in which ERs act as transcription factors within the cell nucleus [4], [5]. These ERs are members of the nuclear receptor superfamily of ligand-modulated transcription factors [6]–[8]. There are two different subtypes of these ERs, referred to as 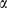 and 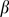, each encoded by a separate gene.

**Figure 1:**
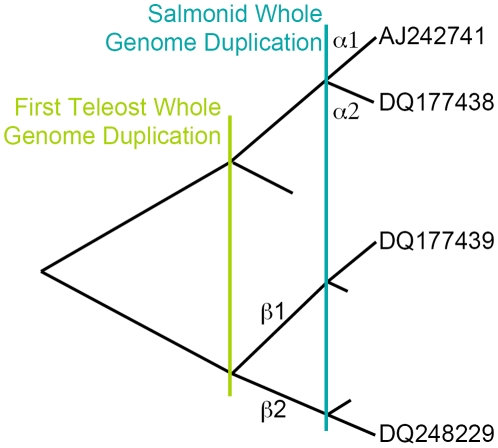
Schematic showing the inferred evolutionary history of the *Oncorhynchus mykiss* estrogen receptors. Vertical bars mark inferred whole genome duplication events; short branches mark inferred duplications that were lost over time.

Recently, Nagler et al [7] reported the novel ER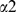 and both ER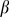 isoforms in the rainbow trout, *Oncorhynchus mykiss*, and performed a comprehensive phylogenetic analysis with all other known fish ER gene sequences. Their phylogenetic analysis indicates that the duplication leading to the two ER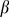 isoforms arose prior to the divergence of the ray finned fish attributable to a whole genome duplication that occurred in the Teleost ancestor (see Figure 1) [9]. The ER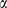 isoforms, on the other hand, appear to have arisen as a result of a second more recent whole genome duplication event that occurred in the salmonid ancestor 25–100 million years ago [10]. These results indicate that the second ER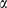 isozyme that arose during the earlier genome duplication appears to have been lost subsequently, since no other ray finned fish are known to have a second ER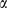 isoform. This also indicates that the expected duplications of ER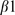 and ER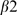 were lost subsequent to the salmonid genome duplication.

The purpose of the study is to employ molecular dynamics simulations to determine the binding affinities between E2 and ERs of the different isoforms in the rainbow trout and to use the results to provide insight into the underlying evolutionary selection pressure on the ERs. Our binding affinity results obtained from insertion and deletion are very similar indicating that our simulations are well converged and that accurate estimates of binding affinities were obtained. Our results show that E2 binds preferentially to ER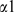 over ER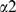. By contrast, the difference in binding affinity is less significant for the 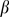 subtype, i.e., both isoforms bind similarly to E2. We also computed 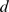
N/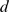
S ratios for the ER isoforms. These results suggest that the ER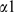 gene is evolving under relaxed selection compared to all other salmonid ER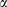 genes.

## Results and Discussion

Experimental binding affinity results are not readily available for the four trout ERs due to the difficulty in isolating the different isoforms. Thus, to verify our methodology for estimating 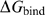 for rainbow trout ER-E2 we first performed simulations using human ER (PDB: 1QKU) at 300 K and compared the binding affinities to the experimental results. Our computational estimates at 300 K are 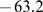 kJ/mol for insertion (when interactions between E2 and its environment are turned on) and 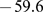 kJ/mol for deletion (when interactions between E2 and its environment are turned off). Experimental binding affinity for human ER is 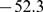 kJ/mol at 300 K [11]. Thus, our human ER binding affinity estimates are within about 10 kJ/mol of experiment which is within the expected error due to the atomic models [12]. The trout ER simulations followed exactly the same procedure as human, beginning with docking the E2 into the ER. It is important to note that our ER simulations were performed at 277 K to closely mimic the water temperature of rainbow trout natural habitat.

Table 1 shows our binding affinity results from both insertion and deletion. Simulation results from both deletion and insertion of electrostatics and Lennard-Jones interactions provide a rudimentary assessment of the accuracy of our calculations (note that there may be inaccuracies in the atomic models but that is beyond the scope of this study). The fact that both insertion and deletion give very similar results strongly suggests that our simulations are well converged and that accurate estimates of binding affinities have been obtained.

**Table 1:** Estrogen receptor binding affinities for different isoforms obtained at 277 K.

(A) Insertion				
	ER 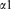	ER 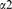	ER 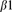	ER 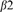
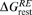	−21.5	−27.6	−31.4	−24.2
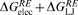	−146.7	−126.5	−132.4	−144.1
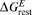	48.9	49.9	52.4	49.9
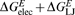	61.8	—	—	—
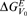	−6.8	—	—	—
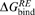	**−64.3**	**−49.2**	**−56.4**	**−63.4**

All results are in kJ/mol. The binding affinities 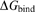 were calculated using Eqn 1. Both insertion and deletion directions give very similar results which demonstrates that our simulations are well converged.

### Evolutionary and Functional Analyses

Our results in Table 1 show that the E2 binds preferentially to the ER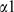 isoform of the 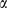 subtype that has been found in all salmonids. The other isoform ER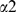, which appears to have arisen during the recent salmonid whole genome duplication, shares 75.4% sequence identity with the ER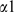 and thus a large number of substitutions have accumulated since the initial duplication event. To infer the evolutionary pressures that led to this amount of divergence in both protein sequence and function, we examined the lineage specific differences in 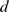
N/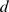
S ratios among the ER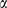 sequences. We used an alignment of the codons in the ligand binding domain for all ER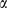 sequences and a phylogeny inferred from the nucleotide sequence by the neighbor joining method (which did not differ significantly from the tree in [7]). PAML was used to calculate the log likelihood values and 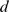
N/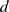
S ratios for each of five hypotheses: a single ratio for all branches, one ratio for all branches except the branch to the rainbow trout ER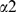, separate ratios for the two ER
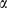
's from rainbow trout and the rest of the tree, separate ratios for the rainbow trout ER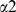, all ER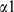 from salmonids and the rest of the tree and the full model where every branch has its own ratio (see Table 2). Using the Aikaike Information Criterion, the model with two ratios, one for the branch to the rainbow trout ER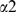 and one for all other branches is the best fitting model. For this model, the 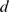
N/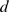
S ratio for all other branches was 0.09 whereas the ratio for the ER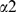 branch was 0.30. In all tests, the 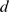
N/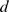
S ratio for the ER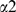 branch was about three times greater than the other salmonid branches. Therefore, the ER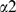 ligand binding domain appears to be evolving under relaxed selection relative to the other salmonid ER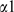 ligand binding domains, which is consistent with the decreased affinity of this domain for E2. It is also possible that ER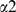 was evolving in a neutral fashion for a short time, but then developed a new function and is now undergoing stronger purifying selection. This possibility could be explored further if more ER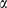 salmonid gene sequences were made available.

**Table 2:** Results of fitting evolutionary models for differences in 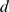
N/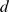
S ratios.

ER 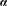 -LBD	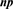	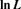	AIC	
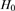 : Everyone is equal	59	−5645.4	11409	
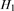 : O 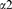 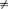 others	60	−5641.7	11403	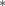
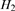 : O 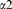 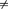 O 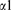 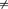 others	61	−5641.6	11405	
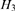 : O 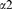 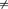 S 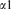 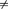 others	61	−5641.5	11405	
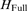 : Everyone is different	115	−5610.3	11451	

LBD and DBD indicate ligand and DNA binding domains, respectively. O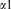, O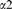, O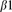 and O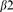 are the *O. mykiss* ER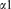, ER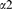, ER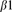 and ER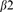 genes, respectively. S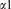 indicates all of the salmonid ER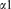 genes. 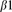 and 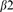 indicate ER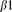 and ER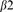 from all fish, respectively. 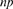 is the number of parameters in the model, 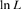 is the log likelihood calculated by PAML, and AIC is the Akaike Information Criterion value [35]. Models labeled with an asterisk are the best fitting models based upon the AIC values.

Our results show that both ER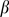 isoforms bind similarly to E2, i.e., the difference between them in binding affinity is small compared to the difference between the ER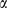 isoforms (see Table 1). The two isoforms share only 57.6% sequence identity, having arisen prior to the Teleost radiation, and the difference in their binding affinity might be expected to be greater, given this large degree of divergence. We performed a similar analysis of the 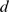
N/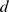
S ratio for these genes by testing the following models: one 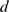
N/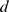
S ratio for the whole tree, a 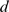
N/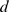
S ratio for each of isoform ER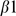 and ER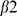, 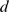
N/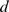
S ratios for each of the two rainbow trout isoforms and for each isoform for all other fish and the full model where every branch has a different 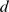
N/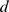
S ratio (Table 2). The best fitting model for this comparison was the single 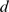
N/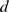
S ratio (0.07) for the entire tree, indicating that both ER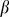 isoforms are under the same level of purifying selection. This is also consistent with our results showing that these two ligand binding domains have similar affinity for E2.

These nuclear ERs have a significant and ubiquitous distribution in the rainbow trout [2], [7]. The levels of transcription differ among the four genes with one isoform having higher transcript levels in most tissues than the other isoform. For the ER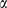 isoforms, ER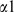 has the higher transcript levels, and for the ER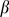 isoforms, ER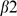 has the highest transcript levels [7]. While the correlation between reduced transcription levels and binding affinity is clear in the ER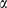 isoforms, there seems to be no such correlation for the ER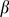 isoforms. These two isoforms share similar binding affinity, and yet, ER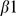 has much lower expression levels than ER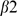 in juvenile rainbow trout. It is possible that both ER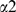 and ER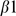 have higher expression levels at other life stages [7]. Given the age of ER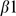 and the equivalent levels of both E2 binding affinity and purifying selection compared with ER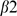, this ER clearly continues to have an important role as an estrogen receptor.

It is not as clear what ER
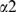
's role is as an estrogen receptor. It's reduced affinity for E2, low transcript levels and evidence for relaxed selection suggests that this estrogen receptor may be undergoing subfunctionalization or neofunctionalization. One indication that ER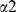 may be undergoing neofunctionalization is that the DNA binding domain of ER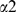 does not have the degree of sequence variation that the ligand binding domain has. If the ER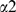 was undergoing relaxed selection along it's entire length, the DNA binding domain would also show indications of greater amino acid divergence (Table 2). It appears that ER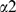 is not losing its ability to bind to the canonical estrogen receptor element even though it is losing affinity for E2. This suggests that this gene may be undergoing neofunctionalization by binding to some other ligand than E2.

### Summaries

Using molecular dynamics simulations we estimated the binding affinities between the hormone 17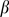-estradiol (E2) and different estrogen receptor (ER) isoforms in the rainbow trout, *Oncorhynchus mykiss*. Our results show that E2 binds preferentially to ER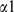 over ER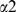. A recent genome wide duplication event led to two functional ER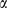 isozymes in *O. mykiss*. Our evolutionary and functional analyses along with Nagler's evaluation of transcription levels [7] suggest that the ligand binding domain of ER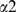 has been or is currently evolving under relaxed selection relative to ER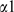. Low sequence divergence of its highly conserved DNA binding domain suggests that ER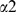 is likely undergoing neofunctionalization, in which it continues to recognize the same estrogen receptor element in the DNA but may be binding to a different ligand. For the ER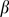 subtype both isoforms bind similarly to E2, in keeping with our evolutionary analyses that both isoforms of this subtype are evolving under the same degree of purifying selection.

## Materials and Methods

### Receptor Structures

**Figure 2:**
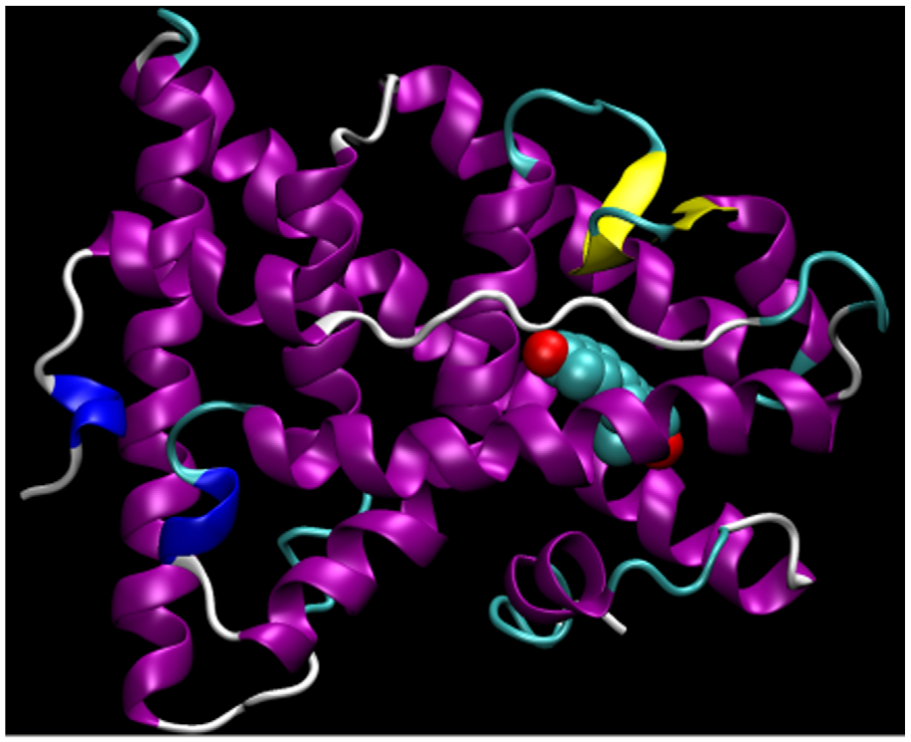
Crystal structure of human estrogen receptor binding domain bound to the hormone 17 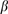
 **-estradiol.** Similar human ER structures were used as templates to generate structures for trout ERs. Image was rendered using VMD [33].

The initial coordinates for the estradiol were first extracted from the human ER-E2 complexes (PDB: 1QKU) (Figure 2). The topologies were then generated by the PRODRG server [13] with the options of full charges and no energy minimization. The rainbow trout ER *holo* structures for the E2 binding domain were generated by SWISS-MODEL [14] using human ER as templates (PDB entries 1A52 for ER
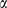
's and 3ERT for ER
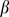
's). Sequence identities between trout and human estrogen binding domains are within the range of 75–85%. The estradiol was first docked into the binding pocket of the receptor *holo* structure with AutoDock [15]. In this protocol, the receptor structure is held rigid and the estradiol is free to rotate and explore most probable binding poses using the Lamarckian genetic algorithm. The number of genetic algorithm runs was set to 1,000 with a population size of 5,000 individuals and 5,000,000 generations. The number of evaluations was set to 2,500,000 for each individual in the population to ensure thorough exploration of the search space. The mutation rate was set to 0.02 and crossover 0.8. Two-point crossover was used to generate the offspring at each successive generation. The genetic algorithm automatically preserved the 10 best-fit individuals to the next generation and the 10 least-fit individuals were not used to generate offspring. A total of 1,000 independent docking trials were performed for each of the four ERs. The best binding pose from each trial was collected and ranked based on the scores. These best-fit binding poses were first visually inspected for consistency with human ER and the one with the highest score was then used as the starting structure for the simulations.

### Thermodynamic Cycle

To estimate E2-ER binding affinities we note that, since the free energy is a state function, it permits the selection of an arbitrary path connecting the bound and unbound states. Therefore, we decomposed the binding free energy calculation into several steps in which the E2 is annihilated (i.e., decoupled) from its bound state in the receptor complex and then made to reappear in solution to complete the thermodynamic cycle. For brevity, we subsequently define *deletion* to be when interactions between E2 and its environment are turned off and *insertion* to be when these interactions are turned on.

**Figure 3:**
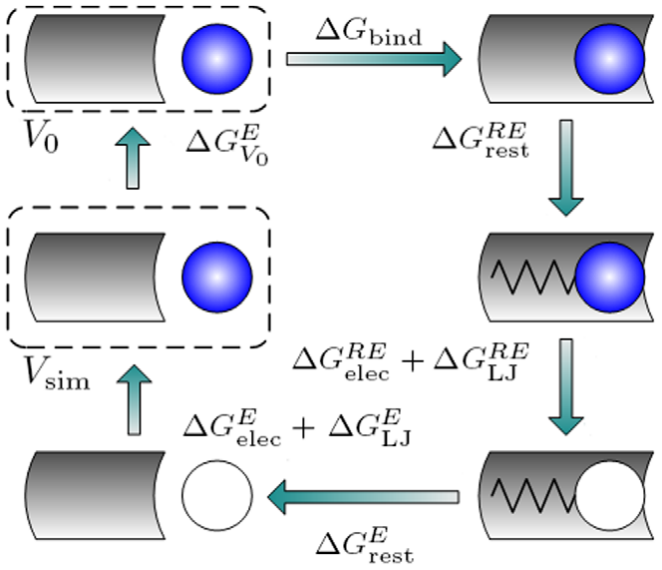
Thermodynamic cycle used for calculating binding affinities. Since the free energy is a state function, the calculation of binding affinity is decomposed into several steps [34]. Eqn 1 was used to calculate the binding affinity 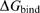 between the ERs and E2. The gray curved rectangle represents the receptor, the blue circle represents E2 with all interactions turned on, and the white circle indicates that all interactions are turned off. The spring represents the restraints between E2 and receptors.

Figure 3 shows the thermodynamic cycle we used to calculate binding affinities (see also Refs [16]–[20]). Starting with upper right schematic and moving clockwise, the fully interacting E2 (blue) is first restrained in the binding pocket of the receptor. Here *RE* represents the solvated complex of the E2 and receptor, and 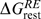 denotes the free energy of restraining the E2 in the binding pocket of the receptor which will depend on the details of restraint. Next, the electrostatic and Lennard-Jones interactions of the E2 are gradually turned off (white) in two separate steps using alchemical simulations. The free energies 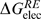 and 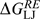 are associated with deleting or inserting electrostatic and Lennard-Jones interactions respectively. With the E2 fully decoupled from its environment, the restraint is then removed. The free energy 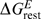 is associated with the removal of this restraint. Next the E2 interactions are turned back on with no receptor present. The free energy 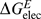 and 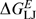 are associated with turning on the electrostatic and Lennard-Jones interactions respectively. Finally, we account for the difference between the standard (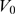) and simulation volume (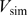). The binding affinity between the estrogen receptors and E2 is thus the sum of the free energies,
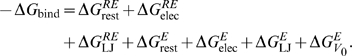
(1)


### Restraints

**Figure 4:**
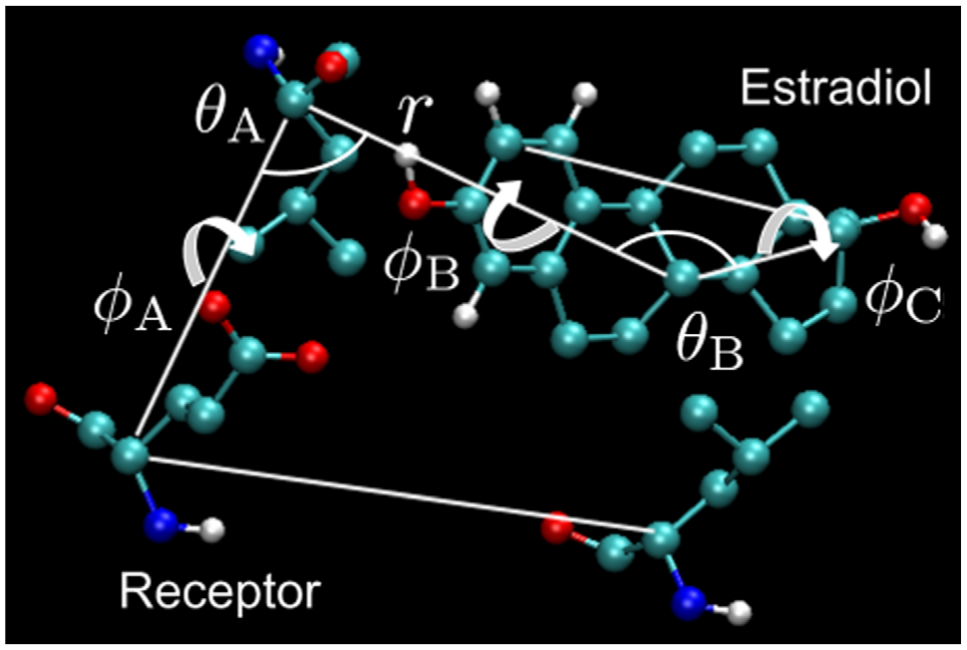
Restraints used for 17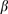-estradiol. We used six harmonic restraints acting on three anchor atoms in the E2 and three in the receptor. The restraints consisted of one distance (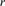), two angle (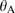 and 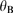), and three dihedral (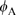, 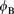, and 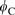) restraints that determine the orientation of three carbons in the E2 relative to three 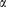-carbons in the receptors.

To facilitate convergence restraints were applied to restrict the positions of E2 relative to the receptors. Boresch et al [21] and Mobley et al [18] reported that the presence of multiple metastable ligand orientations can cause convergence problems for free energy estimates. The authors further suggested using a restraining potential to keep the ligand in the binding site during the simulation process. With such a restraining potential the ligand is no longer required to sample the entire simulation volume (particularly a problem when ligand is decoupled). Moreover, the restraint minimizes the detrimental effects of end-point singularities commonly reported in alchemical simulations [17], [18], [20]. Mobley et al [18] also pointed out that the equilibrium geometry of the restraints is arbitrary and will not affect the asymptotic estimate of the binding free energy. In this work, we judiciously selected anchor atoms from the more rigid alpha helices that form the E2 binding pocket. The restraints included one distance (with the force constant of 1000 kJ/mol/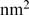), two angle (1000 kJ/mol/rad), and three dihedral restraints (1000 kJ/mol/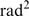) that determine the orientation of three carbons in the E2 relative to three 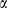-carbons in the receptors (see Figure 4).

### Simulation Protocols

All simulations were performed with the GROMACS 4.0 [12] compiled in single-precision mode at a constant temperature of 277 K in a periodic box with an edge length of approximately 8.2 nm and the default GROMOS-96 43A1 forcefield [22]. The simulation systems each contained approximately 16,500 Simple Point Charge (SPC) water molecules [23]. Short-range interactions were evaluated using a neighbor list of 1.0 nm updated at every 10 steps. Van der Waals interactions used a cutoff with a smoothing function such that the interactions slowly decayed to zero between 0.75 nm and 0.90 nm. A long-range analytical dispersion correction was applied to the energy and pressure to account for the truncation of the Lennard-Jones interactions [24]. Electrostatic interactions were evaluated using the particle mesh Ewald (PME) [25] with a real space cutoff of 1.0 nm, a spline order of 6, a Fourier spacing of 0.1 m, and relative tolerance between long and short range energies of 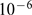. All bonds to hydrogen were constrained with LINCS [26] with an order of 12, and a time step of 2 fs was used for dynamics.

For equilibration, the systems were first minimized using 1,000 steps of L-BFGS (Broyden-Fletcher-Goldfarb-Shanno) [27], followed by 1,000 steps of steepest descent minimization. The system was then subject to 1.0 ns of simulation using isothermal molecular dynamics. This was followed by another 1.0 ns of simulation using isothermal-isobaric dynamics with the Berendsen barostat with a time constant of 1.0 ns. For all simulations the temperature was maintained at 277 K using Langevin dynamics [28] with a friction coefficient of 1.0 amu/ps. The coupling time was set to 0.5 ps, and the isothermal compressibility was set to 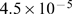 bar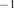.

After equilibration, production simulations were run with isothermal-isobaric conditions using Langevin dynamics at the temperature of 277 K. The pressure was maintained at 1.0 atm using the Parrinello-Rahman algorithm [29]. The temperature was chosen as it closely resembles the water temperature for the natural habitat of rainbow trout. Energies were recorded every 0.2 ps during production runs, and trajectory snapshots every 1.0 ps. The first 50% of each simulation was discarded for equilibration.

### Free Energy Calculations

We used the formula suggested by Boresch et al [21] to analytically calculate the free energy 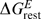 associated with adding the restraints to E2 when decoupled from its environment. We also analytically calculated the free energy 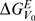 that accounts for the difference between the standard (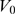) and simulation volume (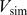) [21].

The free energies 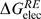, 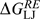, 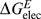, and 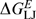, were estimated using the thermodynamic integration (TI) method [16], [19], [20]. To minimize the numerical integration errors we employed the polynomial regression techniques to calculate free energy difference, instead of trapezoidal quadrature [30]. Separate simulations were performed for changes in the Lennard-Jones with 21 values of the scaling parameter, 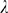 = 0.0, 0.05, 0.1 … 0.9, 0.95, and 1.0, and the electrostatics with 11 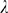 values, 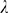 = 0.0, 0.024, 0.095, 0.206, 0.345, 0.5, 0.655, 0.794, 0.905, 0.976, and 1.0. For simulations with only Lennard-Jones, all partial charges were set to zero and the soft-core scaling parameter was set to 0.5. Once the neutral atoms were fully grown in the solvent, the second simulations then computed the free energy associated with the electrostatics with a soft-core scaling parameter of 0.0. This was accomplished by increasing the partial charges from zero to their final values given by the forcefield.

The free energy associated with the restraints, 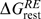 was calculated using the Bennett acceptance ratio approach [31]. We performed 1.0 ns equilibrium simulation for the estradiol-receptor complex using each of the harmonic restraining potentials with force constants of 0, 25, 40, 60, 90, 150, 200, 300, 450, 700, and 1000 kJ/mol/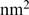 for distance, kJ/mol/rad for angle, and kJ/mol/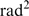 for dihedral restraints. The first 0.5 ns of each simulation was discarded for equilibration and the remaining 0.5 ns was used to compute the free energy differences. No attempt was made to optimize the efficiency of the calculation since our primary objective was to obtain accurate estimates of the restraining free energies.

### Evolutionary Analyses

The following sequences were extracted from GenBank: AB037185, AF349412, A133920050, AY727528, AY775183, BD105560, AB190289, AJ487687, AY055725, AF061275, AF253505, AY520443, AJ242741, DQ009007, DQ248228, DQ177438, X89959, TNU7560, AY422089, AF298183, AF136979, AY074780, AB007453, AJ006039, AF253062, AY223902, ORZMER, AY917147, AF326201, AY305026, NM_180966, NM_174862, AB003356, AB070630, AB070901, AB083064, AB117930, AB190290, AF061269, AF136980, AF177465, AF185568, AF298181, AF298182, AF349413, AF349414, AF516874, AJ275911, AJ289883, AJ314602, AJ314603, AJ414566, AJ414567, AJ489523, AJ580050, AY074779, AY211021, AY211022, AY305027, AY307098, AY508959, AY566178, AY770578, AY917148, BC044349, BC086848, DQ177439, DQ248229, TNU75605. The first 30 are ER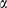 sequences and the other 39 are ER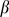 sequences, and the following analysis was done separately for these two subtypes. The codons were aligned based upon their aligned amino acid sequences, and these alignments were used to infer tree topologies using the neighbor joining method. Then the ligand binding domains were extracted from the alignments. PAML was used to test several codon-based likelihood models that allow for variable 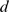
N/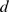
S ratios among lineages based upon the inferred phylogenies and the aligned ligand binding domains [32].
